# Toxic and essential metals: metabolic interactions with the gut microbiota and health implications

**DOI:** 10.3389/fnut.2024.1448388

**Published:** 2024-07-29

**Authors:** Qinheng Zhu, Boyan Chen, Fu Zhang, Baodan Zhang, Yujie Guo, Mengtao Pang, Liang Huang, Tianjiao Wang

**Affiliations:** Department of Personnel Management, Zhejiang Center for Disease Control and Prevention, Hangzhou, China

**Keywords:** toxic heavy metals, essential trace elements, gut microbiota, metabolites, health implications

## Abstract

Human exposure to heavy metals, which encompasses both essential and toxic varieties, is widespread. The intestine functions as a critical organ for absorption and metabolism of heavy metals. Gut microbiota plays a crucial role in heavy metal absorption, metabolism, and related processes. Toxic heavy metals (THMs), such as arsenic (As), mercury (Hg), lead (Pb), and cadmium (Cd), can cause damage to multiple organs even at low levels of exposure, and it is crucial to emphasize their potential high toxicity. Nevertheless, certain essential trace elements, including iron (Fe), copper (Cu), and manganese (Mn), play vital roles in the biochemical and physiological functions of organisms at low concentrations but can exert toxic effects on the gut microbiota at higher levels. Some potentially essential micronutrients, such as chromium (Cr), silicon (Si), and nickel (Ni), which were considered to be intermediate in terms of their essentiality and toxicity, had different effects on the gut microbiota and their metabolites. Bidirectional relationships between heavy metals and gut microbiota have been found. Heavy metal exposure disrupts gut microbiota and influences its metabolism and physiological functions, potentially contributing to metabolic and other disorders. Furthermore, gut microbiota influences the absorption and metabolism of heavy metals by serving as a physical barrier against heavy metal absorption and modulating the pH, oxidative balance, and concentrations of detoxification enzymes or proteins involved in heavy metal metabolism. The interactions between heavy metals and gut microbiota might be positive or negative according to different valence states, concentrations, and forms of the same heavy metal. This paper reviews the metabolic interactions of 10 common heavy metals with the gut microbiota and their health implications. This collated information could provide novel insights into the disruption of the intestinal microbiota caused by heavy metals as a potential contributing factor to human diseases.

## 1 Introduction

**Figure 1:**
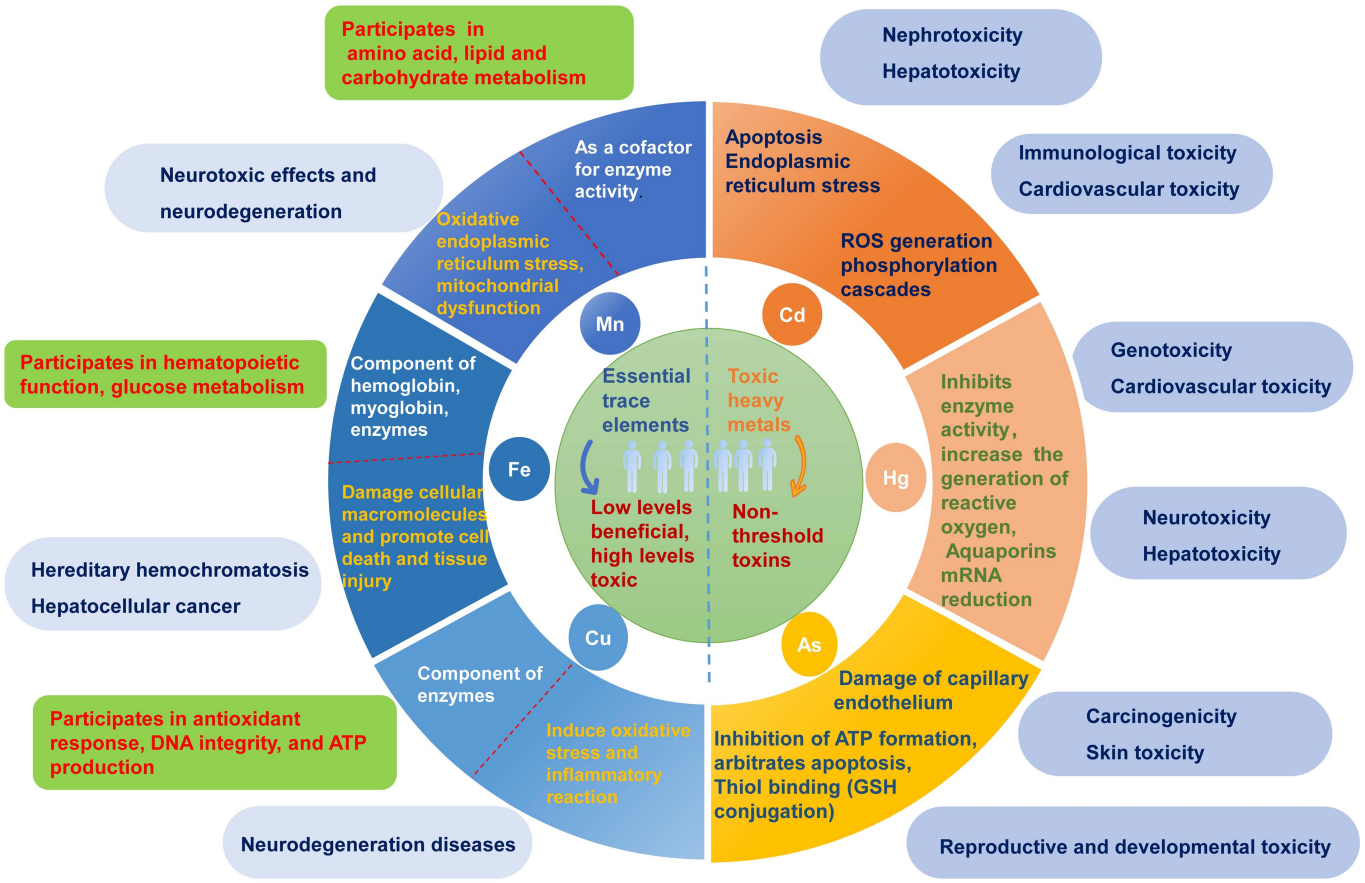
The main physiological functions and potential toxic effects of heavy metals. Heavy metals are categorized into essential trace elements and toxic heavy metals according to whether there is a threshold for toxicity and their physiological functions in the human body. The toxicity and their possible molecular mechanisms are listed on the right side of the circle, while the main physiological functions of essential trace elements and their possible hazards caused by excessive amounts are listed on the left side of the circle.

Heavy metals are commonly defined as elements with densities greater than 5 g/cm^3^ (1). There are over 90 naturally occurring trace elements, with 53 considered heavy metals, primarily transition metals, present in the periodic table. Environmental sources of heavy metals primarily stem from mining, metal processing, and chemical production. These metals enter the human body via inhalation, ingestion, and dermal contact. The toxicity of heavy metals depends on several factors, including dosage, exposure route, chemical species, and individual factors, such as age, gender, genetics, and nutritional status. However, some heavy metals, such as arsenic (As), cadmium (Cd), lead (Pb), and mercury (Hg), are threshold-less toxins known as toxic heavy metals (THMs) that can cause multiorgan damage even at low levels of exposure (2, 3). *In vitro* and *in vivo* studies have shown that the generation of reactive oxygen species (ROS) and oxidative stress play critical roles in the toxicity and carcinogenicity of heavy metals, such as As (4–6), Cd (7), Pb (8, 9), and Hg (3, 10). Conversely, some heavy metals, including iron (Fe), copper (Cu), manganese (Mn), participate in various biochemical and physiological functions at low concentrations, and are considered essential trace elements (11). They are crucial components of several key enzymes that play essential roles in various redox reactions. Trace element deficiencies can lead to various diseases and syndromes. When essential trace elements exceed certain exposure doses, they can exhibit toxic effects. Some potentially essential trace elements, such as chromium (Cr), silicon (Si), and nickel (Ni), are considered to be intermediate in terms of their essentiality and toxicity due to their different forms, concentrations, and valences. The main physiological functions of essential trace elements and toxicity mechanisms of heavy metals, along with their potential toxicity in the human body, are illustrated in Figure 1.

Gut microbiota, often termed the “second genome” of humans, constitutes a major component of the intestinal barrier and is considered a target for heavy metals. Exposure to heavy metals can cause changes in the gut microbiota composition, which may be a vital mediator of heavy metal bioavailability and toxicity. Recently, alterations in gut microbiota induced by exposure to heavy metals and their mediated biological effects have been validated in studies across multiple species, including humans, rodents, and fish (12–16). Moreover, perturbations of the gut microbiota by heavy metal exposure, especially toxic heavy metals, may thus affect metabolic and physiological functions, thereby contributing partly to the development or progression of various injuries and diseases, such as cardiovascular diseases, neurodegenerative diseases, ulcerative colitis, cirrhosis, allergies, diabetes, autism, and inflammatory diseases (17). In fact, a wide array of microorganisms require some essential trace elements to maintain their microbial physiology and viability, as well as the virulence of several pathogenic bacteria, including Salmonella and Escherichia. Deficiency or toxicity of these metals can modulate the gut microenvironment, including the microbiota, nutrient availability, stress, and immunity. The interactions between heavy metals and gut microbiota might be positive or negative according to the different valence states, concentrations, and forms of the same heavy metal. This review summarizes the bidirectional relationship between 10 common heavy metals and gut microbiota, as well as their health implications, providing new insights into heavy metlase-triggered disturbance in the gut microbiota as a potential new causative agent of human diseases.

## 2 Gut microbiota

Gut microbiota refers to the vast microbial community residing in the gastrointestinal tract of host organisms, primarily composed of bacteria, archaea, viruses, and eukaryotes (18). The microbial flora within the human gut consists of bacteria from the phyla Firmicutes, Bacteroidetes, and Actinobacteria, and relatively less abundant members of the phylum Proteobacteria (19). The gut microbiota plays a crucial role in host intestinal epithelial expansion and differentiation, immune regulation, pathogen defense, and maintenance of intestinal homeostasis (20).

### 2.1 Host gut microbiota and its metabolite

Gut microbiota participate in various organismal physiological and pathological functions through extensive interactions with the host via metabolic products (21). Secondary metabolites of gut microbiota include short-chain fatty acids (SCFAs), lipopolysaccharides (LPS), trimethylamine (TMA), branched-chain amino acids (BCAAs), bile acids (BAs), tryptophan, trimethylamine N-oxide (TMAO), and indole (22). LPS, a component released into the intestinal lumen upon bacterial death and lysis, disrupts the host immune and coagulation systems (22). TMA, which is oxidized by flavin-containing monooxygenase 3 (Fmo3) to trimethylamine N-oxide (TMAO), is a key risk factor for cardiovascular and cerebrovascular diseases (23). Bile acids, generated endogenously in the liver and further metabolized by the intestinal microbiota, not only regulate bile acid synthesis but also control bile acid reabsorption, playing a crucial role in human energy metabolism (24). Bile acids can significantly affect host metabolic and immunological processes by activating various bile acid receptors to modulate signaling pathways that regulate a wide array of intricate symbiotic metabolic networks, such as glucose, lipid, steroid, and xenobiotic metabolism, and regulate energy homeostasis (25). Moreover, the gut microbiota can generate essential nutrients, such as vitamins, for the host through bile acid metabolic pathways (26). Indole, a derivative metabolite of the gut microbiota, can strengthen the barrier function of intestinal epithelial cells, inhibit pro-inflammatory cytokine secretion, and reduce host inflammation levels (27). SCFAs, which are metabolites produced by the gut microbiota from carbohydrates that cannot be digested and absorbed by the human body, mainly include acetate, propionate, and butyrate. SCFAs also affect lipid and carbohydrate metabolism by activating acetyl-CoA synthetase in the liver (28).

### 2.2 The bidirectional relationship between heavy metal and gut microbiota

Current researches indicated bidirectional regulation between heavy metal exposure and gut microbiota. Heavy metal exposure can alter the structure, abundance, and diversity of the gut microbiota and influence their metabolic profiles (29). For instance, Pb exposure can significantly increase the ratio of Firmicutes to Bacteroidetes, decrease the total abundance and diversity of gut microbiota, and significantly alter nitrogen metabolism (30). Cd affected the growth of gut microbiota by disrupting protein synthesis and various enzymatic functions (31). Exposure to Cd, Pb, Cu can cause metal-specific and time-dependent changes in the gut microbiota of mice, leading to a significant decrease in the abundance of Akkermansia bacteria (32).

**Figure 2:**
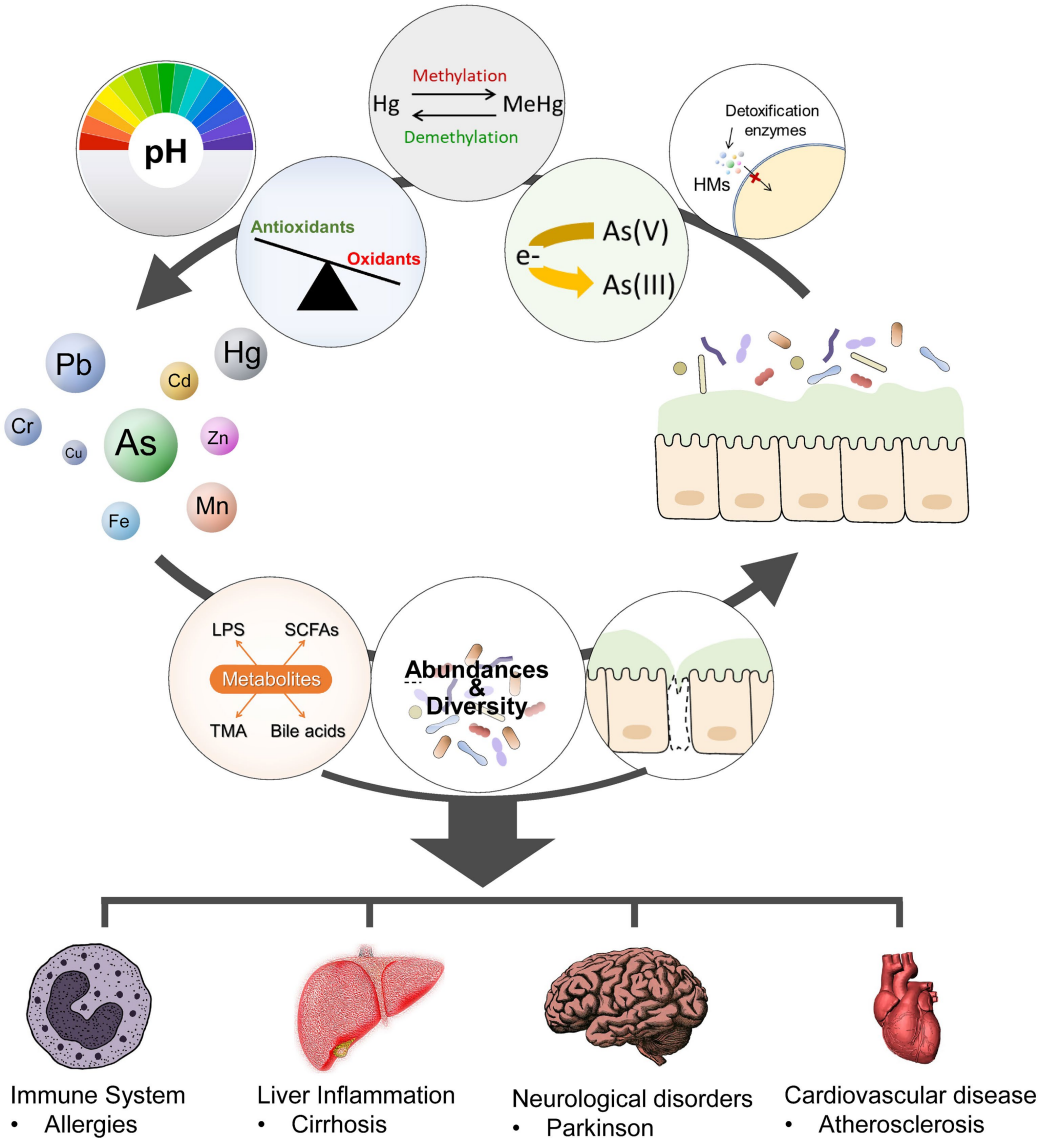
Metal-gut microbiome interactions. The gut microbiota modulate metal toxicity through biotransformation (such as reduction, oxidation, methylations, or demethylations), alter the absorption and metabolism of HMs by modifying the PH and oxidative balance of the gut and the expression of detoxification enzymes or proteins involved in HM metabolism. Heavy metals exposure can alter the composition (abundance, diversity), and metabolic activities of gut microbiota. Dysbiosis and gut barrier disruption may activate the immune system, lead to metabolic and other disorders.

In contrast, the gut microbiota can facilitate the absorption and metabolism of heavy metals by altering the intestinal pH and redox status and modulating the expression of detoxifying enzymes involved in heavy metal metabolism (33). Gut microbiota can bioaccumulate, bind, or transform heavy metals through various enzymatic reactions to promote the excretion of heavy metals, thereby reducing the exposure of organisms to heavy metals (34–36). Iron-binding compounds are chelating molecules of Fe produced by specific gut microbiota such as Pseudomonas, which can also chelate other heavy metal ions (Pb, Cd, Hg, Cr, and As) to form insoluble complexes (37, 38). Similarly, oxalates, the main energy source of the human intestinal symbiotic bacterium Oxalobacter formigens, can bind Pb ions to form insoluble Pb oxalates, thereby reducing Pb absorption (39). Gut microbiota plays an important role in the demethylation of methylmercury, converting it into less toxic and less soluble inorganic forms (40). Gut microbiota can also metabolize As into forms with varying degrees of toxicity through sulfurization and methylation processes (41). One epidemiologic study investigated the changes in diversity and composition profile of gut microbiota resulting from long-term exposure to multiple metals, including As, Cd, Cu, Pb, and Zn. The results showed that long-term exposure to various metals increased the relative abundances of Lachnospiraceae, *Eubacterium eligens*, Ruminococcaceae UGG-014, Erysipelotrichaceae UCG-003, Tyzzerella 3, Bacteroides, Slackia, italics, and Roseburia, whereas the abundance of Prevotella 9 presented an opposite trend. Additionally, differences between male and female groups were found, such as greater richness and evenness of bacteria in men subjected to long-term metal exposure in polluted areas (42). Early postnatal toxic and nutrient element exposures are associated with differences in the infant microbiome (43). A cohort study explored the associations of childhood and perinatal blood metal levels with childhood gut microbiome diversity, structure, species, gene family inferred species, and potential pathway alterations. Long- and short-term associations were found in this study (44). Furthermore, various host factors, including nutrition, gender, age, and immune status, may influence the interaction between gut microbiota and heavy metals. The interactions between the metals and the gut microbiota are illustrated in Figure 2.

The precise mechanism by which the gut microbiota interacts with heavy metals remains unclear. The major mechanism of this interaction might be through influencing the production and absorption of metabolites, which have regulatory roles in immunological maturation, immune homeostasis, host energy metabolism, and mucosal integrity preservation (45). For example, an As-induced increase in LPS can activate Toll-like receptors (TLR) 4 and 2 and LPS receptor CD14, leading to increased activation of inflammatory pathways; such activation then causes impairment of insulin signaling, which can lead to diabetes (46). In addition, a growing number of studies have demonstrated that probiotics are effective against heavy metals and pathogens by maintaining gut barriers and healthy gut bacterial counts (47). Lactobacillus and Bifidobacterium were the most important species studied, followed by Faecalibacterium and Akkermansia (48). Several other types of probiotics, including Bacillus, Lactococcus, *Faecalibacterium prausnitzii*, and *Akkermansia muciniphila*, as well as genetically modified organisms, have emerged as promising probiotic candidates. These are often referred to as “next-generation probiotics.” Probiotics are HM-binding and have various beneficial properties for HM detoxification and bioremediation, (such as high tolerance to gastrointestinal fluid, strong adhesion to the intestinal mucosa, potent suppression of pathogen growth, and strong antioxidative and immunoregulatory capacities) (49). For example, studies have shown that dietary Lactobacillus spp. and Bacillus spp. can significantly reduce the absorption of a single heavy metal (e.g., Cd, Cu, or As) in animals (e.g., fish and crayfish). The aforementioned bacterial cells can mitigate the toxic consequences by preserving the integrity of the gut barrier, enhancing the expression of tight junction proteins, stimulating the production of epithelial cells for macrophages and anti-inflammatory responses, and maintaining the abundance and diversity of the gut microbiome (50, 51).

### 2.3 The methods for determining the relationship between heavy metals and gut microbiota

The relationship between gut microbiota and heavy metals was primarily examined using *in vivo* and *in vitro* assays. Various animal models, along with clinical trials, have been employed to explore the impact of heavy metals on the diversity and composition of the gut microbiota. For example, oral antibiotic-treated (AT) or germ-free (GF) mice have been widely used to explore the function of gut microbiota in heavy metal absorption and metabolism (49). *In vitro* gut fermentation models that can simulate the physiological environment of the gut, including pH, temperature, and anaerobic conditions, were applied to explore this relationship. Simulated human intestinal microbial ecosystem (SHIME) was also used to demonstrate the critical role of the human gut microbiota in the transformation and metabolism of As (52, 53).

## 3 Gut microbiota and toxic heavy metals

Toxic heavy metals persist in the environment and are difficult to degrade, leading to potential environmental pollution issues owing to human industrial and lifestyle activities. The effects of toxic heavy metals on the gut microbiota and metabolism have been extensively studied in terms of their potentially high toxicity. The exposure of animals to As, Cd, Pb, and Hg results in alterations in the gut microbiota composition and metabolic profile, thus inevitably damaging gut integrity and amplifying the resulting host microbiota signals, leading to a series of downstream effects. As-induced alterations in gut microbiota may lead to further changes in murine physiology, such as changes in nutrient absorption and fat distribution, weight loss, diabetes, and innate immune responses (54). Pb-mediated changes in gut microbiota may contribute to weight gain and other related diseases (55). The bidirectional relationships between THM and gut microbiota, as well as THM-induced changes in gut microbiota metabolites, are shown in Table 1.

**Table 1:** The relationship of toxic heavy metals and gut microbiota as well as heavy metal-induced changes in metabolites.

Heavy metals	Experiment model	Effect on gut microbiota composition	Effect on gut metabolite and gut barrier	Outcome	References
As	Tilapia	↑ Stenotrophomonas	_	Transform As(V) to As(III) and organoarsenicals	(56)
As	Mice	↑ Bacteroidetes ↓ Firmicutes	Changed major unconjugated primary bile acids and decreased secondary BAs in the serum and liver	Perturbed Bile Acid Homeostasis	(57)
As	Mouse pups	↑ Akkermansia, Bacteroides ↓ Muribaculaceae	Increased intestinal permeability and inflammation	Amino acid and purine metabolism was promoted glycerophospholipid metabolism was inhibited	(58)
As	Mice	↑ Clostridium, sulfatireducen, Actinobacteria, Bacteroidetes, Alistipes, johnsonii, Butyricicoccus, Parasporobacterium, Bilophila, Phyllobacterium, Deferribacteres, *Mucispirillum schaedleri* ↓ Intestinimonas	Increased chemokines and pro−/anti-inflammatory cytokine secretion in intestine of adult mice	Metabolize As into less-reactive trivalent changes of intestinal mucosal immune cytokines induced by As maybe a mechanism to prevent arsenic toxicity	(59)
As	Mice	↑ Bacteroidetes, Alkalitalea, Chryseobacterium, Alistipes ↓ Tenericutes, Anaeroplasma, Clostridium_XlVb, Syntrophococcus, Fusicatenibacter, Cellulosilyticum	Affected BAs and Amino acids metabolism	Changed gut microbiome and metabolome by impacting bile acids, amino acids and several gut bacteria associated with metabolic health	(60)
As	Mice	↑ Bacteroidetes, Bacteroides, Porphyromonadaceae, Barnesiella, Lactobacillus, Nitrite reducing bacteria ↓ Firmicutes, Lachnospiraceae, Ruminococcaceae,	Promoted nitrogen and amino acid metabolism	As induced changes in gut microbiota may cause further changes in nutrient uptake and fat distribution Accelerated aging, weight loss, diabetes and innate immune responses	(54)
Hg	Mice	↑ Coprococcus, Oscillospira, Helicobacter ↓ Lgnatzschineria, Salinicoccus, Bacillus	Increased the expression of pro-apoptotic genes including Bax, JNK, ASK1, caspase3 and TNF-alpha, and significantly decreased the expression of the anti-apoptotic gene Bcl-2.	Hg exposure prominently effected body weight gain and glucose levels Hg exposure induced intestinal microbiota dysbiosis and metabolic disorder, and aggravated apoptosis in mice.	(61)
Hg	Rats	↑ Eubacteriaceae, Porphyromonadaceae, Bdellovibrionaceae, Rikenellaceae, Erysipelotrichaceae ↓ Lactobacillaceae, Peptostreptococcaceae, Streptococcaceae	Ntestinal cell necrosis occurred in the small intestine wall and large intestine wall and the intestinal mucosa was destroyed	IHg also changed gut-liver axis related metabolites	(62)
Hg	Rats	↑ Firmicutes, Peptococcaceae, Desulfovibrionaceae, Helicobacteraceae, Rhodospirillaceae ↓ Lactobacillaceae, Streptococcaceae, Bacteroidetes, Bacteroidaceae, Proteobacteria, Sutterellaceae	MeHg exposure affected metabolic profiles in feces, including the metabolism of nucleotide, carbohydrate, amino acid, and lipid, and intestinal immune system.	Acute oral MeHg exposure perturbs the gut microbiota and alters gut-brain related metabolites, Damages the intestinal integrity and induces inflmmatory, immunological and neurological responses.	(63)
Hg	Mice	↑ Butyricimonas, Dehalobacterium, Bilophila, Coprococcus, Oscillospira ↓ Sporosarcina, Jeotgalicoccus, Staphylococcus, Acinetobacter	_	Decreased body weight Caused histopathological lesions in the cecum Altered gut microbiota	(64)
Hg	Fish	↑ Bacteroidetes, Bacteroidales, Cloacibacterium, Proteobacteria: Xanthomonadaceae ↓ Acinetobacter, Xiphinematobacter, Aeromonas, Comamonadaceae families, Pseudomonas, Nocardia, Deltaproteobacteria	MeHg disturbed lipid metabolism and neurotransmission	Dietary MeHg exposure alterted intestinal microbiome and metabolome in *Pimephales promelas*	(65)
Pb	Mice	↓ Ruminococcus, Coprococcus, Oscillospira, Blautia	Reduced vitamin E and BAs, altered the nitrogen and energy metabolism, induced oxidative stress and activated detoxification mechanism	Altered gut microbiota structures/diversity Greatly affected metabolic functions	(29)
Pb	Human	↑ Succinivibrionaceae, Enterococcaceae, Eubacterium, Ruminococcus, Alcaligenaceae, Barnesiellaceae, Rikenellaceae, Proteobacteria, Brucellaceae, Desulfovibrionaceae, Oxalobacteraceae, Desulfovibrio ↓ Clostridiaceae, Lactobacillaceae, Clostridium, Coprococcus, Pediococcus	_	Increasing urinary Pb levels were associated with increases in microbial α-diversity and richness	(39)
Pb	Mice	↑ Parabacteroides, Proteobacteria ↓ Dehalobacterium	Chronic Pb exposure affected amino acid metabolism, the TCA cycle, energy metabolism in gut microbiota.	Chronic Pb exposure at low level increased hepatic TG and TCH levels Induced dysbiosis of the gut microbiota and metabolic disorder	(66)
Pb	Budgerigars	↑ Firmicutes, Proteobacteria, and Bacteroidetes	Phenylpropanoids and polyketides, Organoheterocyclic compounds, Organic oxygen compounds, and Organic nitrogen compounds were dominant metabolite superclasses.	Pb exposure increased liver weight Decreased body weight Cell apoptosis was increased	(67)
Pb	Mice	↑ Firmicutes/Bacteroidetes levels, Erysipelotrichaceae ↓ Rikenellaceae, Akkermansiaceae, Ruminococcaceae	Decreased SCFAs levels in HFD mice	Exacerbated hyperglycemia and insulin resistance Impaired glucose tolerance in HFD mice Increasing intestinal permeability and inflammation	(68)
Cd	Mice	↑ Prevotella, Barnesiella, Parabacteroides, Alistipes, Alkalitalea ↓ Desulfovibrio, Klebsiella, Parasutterella, Acinetobacter, Clostridium_XlVb, Syntrophococcus, Cellulosilyticum	Affected the BAs and amino acids metabolism	Changed metabolic health-associated gut microbiome and metabolites	(60)
Cd	Freshwater crayfish	↑ Azoarcus, Bacteroides, Shewanella, Anaerorhabdus, Alistipes, Chryseobacterium, Fusobacteria ↓ Hafnia, Buttiauxella, Arcobacter	Up-regulated metabolism of amino acid, carbohydrate, energy, and nucleotide, while up-regulated enzyme families, lipid metabolism, and xenobiotics biodegradation and metabolism	Affected intestinal microbiota composition and functions Induce intestinal histological damage	(13)
Cd	Mice	↑ Helicobacter, Campylobacter	Activated intratumoral glutamate metabolism	Enhanced mammary tumorigenesis	(69)
Cd	Bees	↑ Bacterial diversity ↓ Fungal diversity	Cause an increase in malondialdehyde content. The transcription of immune-related genes and acetylcholinesterase activities was inhibited	Increased the mortality Reduced the number of transcripts of antioxidant genes and superoxide dismutase activity	(70)
Cd	Rats	↑ *Escherichia coli*_Shigella ↓ Prevotella, Lachnoclostridium	Serum TG, LPS, and inflammatory cytokines were increased with the expressions of IL-1β, IL-6, TNF-α genes up-regulated in liver and kidney	Cause disturbance of gut microbiota, dysfunction of liver, kidney, and ovary Inflammatory response, dyslipidemia, kidney dysfunction, and abnormal estrogen level	(71)

↑, higher/increased; ↓, lower/decreased; —, Not applicable; HFD, high-fat diet; As, Arsenic; Hg, mercury; Pb, lead; Cd, cadmium; TNF-a, tumor necrosis factor-alpha; IHg inorganic mercury; MeHg, methyl mercury; TCA, tricarboxylic acid; TG, triglyceride; TCH, total cholesterol; LPS, lipopolysaccharide.

### 3.1 Arsenic (As)

The toxicity of As is determined by its various chemical forms and oxidation states. Trivalent arsenic (e.g., iAs^III^, MMA^III^, and DMA^III^) exhibits higher toxicity than pentavalent arsenic (e.g., iAs^V^, MMA^V^, and DMA^V^). As poisoning primarily arises from exposure to contaminated drinking water and food. Song et al. (56) first observed that the intestinal microbiota of tilapia could convert inorganic As into less toxic organic forms. Exposure to As(V) significantly altered the distribution of fish intestinal microbial communities, with Burkholderia as the predominant genus. As exposure reduced the alpha diversity of the intestinal microbiota, with a notable increase in the relative abundance of Bacteroidetes and a significant decrease in Firmicutes (57). As exposure disrupts bile acid metabolism in mice by modulating the expression of various bile acid receptors including farnesoid X receptor (FXR), pregnane X receptor (PXR), vitamin D3 receptor (VDR), constitutive androstane receptor (CAR), and G protein-coupled bile acid receptor 1 (TGR5) (72). For example, FXR, primarily expressed in the liver and intestine, regulates the expression of genes related to bile acid synthesis, absorption, and excretion and serves as a key regulator of bile acid homeostasis (73). Thus, the induced dysfunction of bile acid metabolism may represent one of the potential mechanisms underlying arsenic toxicity in the host. Developmental exposure to As has been found to induce dysbiosis of the intestinal microbiota and disturbances in plasma metabolites in mice (58). Methylated trivalent arsenic such as MMA^III^ and DMA^III^ can reduce glucose-stimulated insulin secretion (GSIS) by impairing calcium influx stimulated by glucose, thereby playing a significant role in diabetes (74). The gut microbiota influences the absorption pattern of arsenic. Research has indicated that, when contrasted with mice possessing dysbiotic gut microbiota, conventional mice display lower levels of total arsenic and MMA/DMA ratios in their urine. It has been observed that alterations in the ratio of MMA to DMA in urine are linked to a heightened risk of several ailments (75), including skin cancer and skin lesions, as per previous studies (76, 77). Higher MMA levels are associated with increased risk of cancer and cardiovascular diseases, whereas lower MMA ratios are associated with increased risk of diabetes and metabolic syndrome (78). The existence of the gut microbiome can alter the expression of genes related to As metabolism in the host. Moreover, trivalent species have been found to inhibit the activity of 3-phosphoinositide-dependent kinase-I (PDK-1) and mammalian target of rapamycin (rictor-mTOR), and to cause decreased phosphorylation of Akt, resulting in decreased GLUT4 recruitment to the plasma membrane of insulin-stimulated adipocytes. Akt is important for GLUT4 translocation, and the lack of GLUT4 recruitment inhibits glucose uptake and causes hyperglycemia (79).

### 3.2 Mercury (Hg)

Hg exists primarily in three forms in the environment: elemental mercury (Hg^0^), inorganic mercury (Hg^+^ and Hg^2+^), and organic mercury (methylmercury, ethylmercury, and phenylmercury) (80). The toxicity of Hg is primarily attributed to oxidative damage and its binding to thiol groups within cells (10). Human exposure to Hg arises mainly from the consumption of mercury-contaminated foods, such as fish and rice, or mercury-containing medications. Numerous studies have highlighted the crucial role of gut microbiota in the demethylation of methylmercury (MeHg), converting it into less toxic, less soluble inorganic forms within organs (81). Investigations employing antibiotic treatment (AT) and germ-free (GF) mice have explored the role of the gut microbiota in heavy metal absorption. The results indicated a significant reduction in methylmercury excretion and higher concentrations accumulated in the blood and tissues of AT and GF mice, suggesting that the gut microbiota plays a pivotal role in reducing heavy metal absorption and accumulation. Oral exposure to methylmercury disrupts the gut microbiota and induces neurotoxicity via the gut-brain axis (65) and even hepatocarcinogenesis via the gut-liver axis (62).

Hg exposure can alter the structure and abundance of the gut microbiota. At the phylum level, Hg^2+^ increased the abundance of Firmicutes, Bacteroidetes, and Actinobacteria, but decreased the abundance of Proteobacteria and Verrucomicrobia (62). At the family level, Hg^2+^ exposure increased the relative abundance of Enterobacteriaceae and Pseudomonadaceae (82). In mice poisoned with Hg^2+^, significant alterations in pathogenic bacteria such as Streptococcus, Enterococcus, Staphylococcus, *Streptococcus pneumoniae*, Corynebacterium, *Bacillus anthracis*, and Helicobacter have been observed in fecal samples (62). Due to the consumption of seafood and fish, individuals are more likely to be exposed to the more toxic methylmercury compared to inorganic mercury, and the intestinal absorption rate of MeHg is significantly higher than that of IHg. Exposure to methylmercury increases the abundance of Proteobacteria and Bacteroidetes but decreases the abundance of Desulfobacterota at the phylum level (65). At the family level, methylmercury exposure increases the abundance of Pseudomonadaceae, Prevotellaceae, and Neisseriaceae but decreases the abundance of Lactobacillaceae in the intestine (82). Animals exposed to high doses of IHg exhibit a significant decrease in Shannon and Simpson indices, indicating reduced diversity of the gut microbiota following IHg exposure (83).

Oral exposure to methylmercury disrupts intestinal metabolism. The gut microbiota participates in the production of many neurotransmitters, such as serotonin, dopamine (DA), and other metabolites, such as creatine, serine, L-threonine, and N-acetylserotonin (63). Therefore, dysbiosis of the gut microbiota following methylmercury exposure affects the secretion of neurotransmitter-related metabolites (84). The levels of glutamate in fecal samples from fish and mice exposed to methylmercury were significantly increased (65). γ-Aminobutyric acid (GABA) is an inhibitory neurotransmitter formed by the decarboxylation of glutamate (85) and was found to be significantly elevated in the feces of methylmercury-intoxicated rats (63). The abundance of Bacillus, Rhodococcus, and Acinetobacter increases in mice exposed to MeHg (86, 87), which affects the production of short-chain fatty acids (SCFAs). Short-chain fatty acids participate in neuronal circuits in the brain and serve as important signaling molecules in the gut-brain axis (63). Furthermore, one study found that changes in certain bacteria in the intestine, such as Aeromonas and Acinetobacter, inhibit the immune function of fish (65), indicating that MeHg may indirectly disrupt intestinal mucosal immune responses through the gut microbiota, leading to the occurrence of various immune diseases (88). Significant relationships were observed between Hg concentrations and the abundance of Parabacteroides and Oscillospira in the guts of children with autism (89). These findings suggest that Hg metabolism and perturbed gut microecology contribute to the pathogenesis of autism spectrum disorders.

### 3.3 Lead (Pb) and cadmium (Cd)

Numerous studies have confirmed that the potential health damage induced by Pb and Cd exposure has emerged as a global public health concern. Gao et al. found that Pb exposure reduced the α-diversity and richness of the gut microbiota (29). Metabolomic analysis results showed that Pb exposure significantly disrupted various metabolic pathways of the mouse gut microbiota, including vitamin E, bile acids, nitrogen metabolism, energy metabolism, oxidative stress, and defense/detoxification mechanisms. However, contrasting results were observed in another study (39). The reasons for the inconsistency in the above results may be: (1) Pb exposure leads to increased viability of some newly formed bacteria, (2) Pb exposure reduces the abundance of high-abundance bacteria, thereby altering the relative proportions of other bacterial species, and (3) the immunosuppressive effect of Pb outweighs its direct effect on the gut microbiota, thereby promoting the colonization of gut microbiota in the intestines. Bisanz et al. found that elevated blood Pb levels were associated with an increased relative abundance of Succinivibrionaceae and Gammaproteobacteria, thereby promoting Pb absorption (90). Bgki77x1 Histopathological and immunofluorescence analysis results show that lead treatment alters the metabolites of the gut microbiota, leading to increased apoptosis and significant liver damage (67). Pb exposure changes the structure of the gut microbiota in high-fat diet (HFD) mice, increasing the levels of Firmicutes/Bacteroidetes in HFD mice, increasing the abundance of Erysipelotrichaceae at the family level, and decreasing the abundance of Rikenellaceae, Akkermansiaceae, and Ruminococcaceae, while also reducing the levels of SCFAs, leading to imbalances in glucose homeostasis, intestinal mucosal damage, and inflammation (68). Wu et al. (54) found that prenatal exposure to Pb could result in a decrease in Akkermansia and an increase in Desulfovibrio, which in turn could contribute to an increase in body weight in adult male mice. Studies have shown that Akkermansia has the potential to improve adiposity, whereas Desulfovibrio can convert choline into trimethylamine (TMA). TMA can then be further oxidized in the liver to form TMA N-oxide (TMAO), a compound strongly linked to the development of cardiovascular disease and colon cancer (91).

Zhang et al. found that Cd exposure alters the abundance, diversity, and composition of the gut microbiota in freshwater crayfish. It increases the metabolism of energy, amino acids, carbohydrates, and nucleotides in crayfish, while decreasing lipid metabolism and exogenous biodegradation and metabolism (13). Xu et al. found that disruption of the gut microbiota in zebrafish exacerbated Cd-induced neurotoxicity, which may be related to the expression of several genes in the V-ATPase enzyme family (92). Cd exposure disrupts intestinal microbiota homeostasis in mice, especially by increasing the abundance of Helicobacter and Campylobacter, activating glutamate metabolism, and promoting the occurrence of breast tumors (69). Cd exposure increased bacterial diversity in bee intestines, decreased fungal diversity, and increased bee mortality, disrupting redox balance, increasing oxidative stress, and downregulating immune genes (70). Adolescent rats exposed to Cd experience gut microbiota dysbiosis, significantly reducing the abundance of Prevotellaceae and Lachnospiraceae, increasing the abundance of *Escherichia coli* and Shigella, leading to increased levels of serum triglycerides (TG), LPS, and inflammatory factors, and upregulating the gene expression of IL-1β, IL-6, and TNF-α in the liver, kidneys, and ovaries, resulting in dysfunction of the liver, kidneys, and ovaries (71). Disruptions in energy metabolism induced by Pb and Cd may be related to the decreased abundance of Firmicutes and increased abundance of Proteobacteria and Bacteroidetes (66). Furthermore, Pb and Cd exposure affects the concentration of SCFAs by altering the abundance of bacteria capable of biosynthesizing SCFAs, such as Ruminococcus, Bacteroides, and Oscillospira (93, 94). Ba et al. found that early-life low-dose cadmium exposure induced changes in the gut microbiota, liver lipid metabolism disorders, and fat accumulation in mice in a gender-dependent manner (95).

## 4 Interactions between different essential trace elements and gut microbiota

Serveral essential trace elements such as Mn and Fe are critical for maintaining the survival and fitness of gut microbiota by regulating the function of proteins involved in the detoxification of reactive oxygen species, metabolism of carbohydrates, lipids, and proteins, and DNA replication. For instance, certain gut bacteria and pathogens, such as *Escherichia coli*, Yersinia species, and *Salmonella enterica*, have demonstrated the ability to transport, metabolize, and utilize trace elements to sustain survival. The deficiency or toxicity of these essential trace elements can modulate the intestinal microenvironment, including microbial community structure, nutrient availability, stress response, and immune response. Zhang et al. found that Cu, while regulating the gut microbiota in pigs, also altered energy and protein metabolism, as well as amino acid biosynthesis (96).

Certain bacteria can maintain Cu homeostasis by regulating its transportation and translocation, thus preventing its toxic effects (97, 98). Iron regulates bacterial survival through several key metabolic pathways, including riboflavin biosynthesis, antioxidant enzyme function (i.e., catalase), anaerobic respiration, butyrate production, and pathogen virulence, indicating that iron availability is tightly regulated in the gut and plays a crucial role in maintaining a healthy microbial community (99–101). Both deficiency and excess of trace metals, such as iron, manganese, zinc, and copper, contribute to the onset and progression of metabolic disorders, pathogenic overgrowth, and immunological dysfunction. The bidirectional relationships between essential trace elements and gut microbiota, as well as element-induced changes in gut microbiota metabolites, are shown in Table 2.

**Table 2:** The relationship of essential trace elements and gut microbiota and elements-induced changes in metabolites.

Trace elements	Experiment model	Effect on gut microbiota composition	Effect on gut metabolite and gut barrier	Outcome	References
Cu	Rats	↓ Probiotics, the ratio of Firmicutes to Bacteroidetes (F/B), Changed the abundance of fat metabolism and intestinal inflammation-related bacteria.	Phenylalanine and tyrosine metabolism, catecholamine biosynthesis, thyroid hormone synthesis, purine metabolism, amino sugar metabolism, and arachidonic acid metabolism, were significantly affected	Induced liver damage and gut microbiota dysbiosis and affected the relevant metabolic pathways	(102)
Cu	Juvenile carp	The ratio of Bacteroidetes to Firmicutes was increased	Cu2+ could induce significant changes in MDA content and antioxidant enzyme SOD, CAT, glutathione peroxidase GPx activity	Cause histological alterations such as structural damage to the liver and intestines	(103)
Cu	Juvenile common carp	↑ Pseudomonas, Acinetobacter ↓ Allobaculum, Blautia, Coprococcus, Faecalibacterium, Roseburia, Ruminococcus, Lactobacillus, Bacillus, Akkermansia	Roseburia sequences were positively associated with lipogenic enzymes, TP, and TGs and negatively associated with lipolysis enzymes.	Exposure to a high concentration of Cu had a negative effect on growth indices WGR and SGR. The biochemical indices measured in serum LDL and TGs were significantly affected by exposure to medium concentration levels of Cu.	(104)
Cu	Piglets	↑ Streptococcus ↓ Roseburia, Acidaminococcus, Coprococcus, Lachnospiraceae	Heptanoic acid was increased, carbohydrate, amino acid metabolism was decreased	↓ TNF-α, SOD, serum albumin	(96)
Cu	Pig	↑ Proteobacteria, Spirochaetes ↓ Firmicutes, Bacteroidetes	↑ Valine, leucine isoleucine, lysine biosynthesis; lipid biosynthesis ↓ Peptidases	Affected the microbial metabolic functions of energy metabolism, protein metabolism, and amino acid biosynthesis	(105)
Mn	Mice	Male mice: Firmicutes↑ Bacteroidetes↓ Female mice: Firmicutes↓ Bacteroidetes↑	The abundances of LPS biosynthesis genes were widely increased in Mn2+ treated female mice, but reduced in male animals	Altered the abundance of bacterial genes and GABA metabolism pathways Aphla-tocopherol and gama-tocopherol were decreased in Mn2 + −treated male and female mice	(106)
Mn	Rats	↓ Prevotellaceae, Fusobacteriaceae Lactobacillaceae	Mn exposure altered the metabolism of tryptamine, taurodeoxycholic acid, β-hydroxypyruvic acid and urocanic acid	Increases host manganic bioaccumulation, and β-amyloid (Aβ), receptor-interacting protein kinase 3 (RIP3) and caspase-3 production in the brain Causes hippocampal degeneration and necrosis	(107)
Mn	Geese	↑ Bacteroidetes, Bacteroidaceae, Bacteroides, Ruminococcaceae ↓ Streptococcaceae	Enhanced the total antioxidant capacity by increasing the activity of total superoxide dismutases or decreasing the content of malondialdehyde.	Decrease the DFI of geese Increased the laying rate Elevate the hatching egg hatching rate and eggshell thickness	(108)
Mn	Male rats	_	Mn exposure stimulates neurotoxicity by increasing inflammation either in peripheral blood and CNS.	Increases Aβ1-40 and Tau production in brain Causes hippocampal degeneration and necrosis	(109)
Fe	Mice	↑ Romboutsia, Erysipelatoclostridium Akkermansia, Bifidobacterium, Lactobacillus	Serum total cholesterol and TG levels were both reduced	Decrease in body weight gain, body fat and lipid accumulation of liver	(110)
Fe	Piglets	↑ Lactobacillus, Roseburia ↓ Veillonella, Escherichia-Shigella, Actinobacillus, Streptococcus, Fusobacterium	↓ IL-1β, TNF-α, urinary lactulose-to-mannitol ratio ↑ disaccharidase activity	Diarrhea incidence decreased Intestinal villi height increased	(111)
Fe	Mice	↑ Verrucomicrobiota, Allprevotella, Muribaculum ↓ Dubosiella, Lactobacillus, Bifidobacterium	Iron overload reduces the concentration of metabolites which exert the anti-inflammatory effects in mice	Iron overload induces colitis by activating ferroptosis of colonic cells and interfering gut microbiota in mice	(112)
Fe	Pigs	↑ Bifidobacterium, Dialister, Prevotella, Lactobacillus, Megasphaera ↓ Bacteroides, Clostridium, Akkermansia, Clostridiaceae, Lachnospiraceae, Ruminococcaceae,	The total volatile fatty acid concentration was higher in iron deficiency pigs compared with control pigs in both ascending colon and feces but no differences in VFA concentrations persisted to postnatal day	Early-life iron status influenced microbial composition and VFA concentrations within the large intestine, but these differences were largely normalized following subsequent dietary iron repletion.	(113)
Fe	Rats	↑ Lactobacillu, Enterobacteriaceae ↓ Roseburia, Bacteroides, *Eubacterium rectale*	In plasma and liver of normal and growth-restricted rats, excess iron increased 3-hydroxybutyrate and decreased several amino acids, urea and myo-inositol.	Excess iron adversely affects cognitive development	(114)

↑, higher/increased; ↓, lower/decreased; —, Not applicable; MDA, malondialdehyde; Cu, Copper; Mn, manganese; Fe, Iron; SOD, superoxide dismutase; CAT, catalase; GPx, glutathione peroxidase; TP, total protein; TGs, triglycerides; WGR, weight gain rate; SGR, specific growth rate. LDL, low-density lipoprotein; TNF, tumor necrosis factor; LPS, lipopolysaccharide; GABA, γ-aminobutyric acid; DFI, the daily feed intake; CNS, central nervous system; IL, interleukin; VFA, volatile fatty acids.

### 4.1 Copper (Cu)

Cu is an essential element that serves as a cofactor for various enzymes involved in antioxidant reactions (e.g., superoxide dismutase), biological membrane and DNA integrity, and ATP production. The widespread use of Cu in industry, mining, and agricultural production is a major source of Cu contamination in soil and water. Recent research suggests that high-cholesterol-induced Cu deficiency may regulate pig gut microbiota by increasing the abundance of Enterobacteriaceae and Vibrionaceae and decreasing the abundance of Bacteroidaceae and Ruminococcaceae (115, 116). Increasing evidence suggests that adding more copper to feed may increase opportunistic and pathogenic bacterial resistance and decrease the abundance of several probiotics (117). Dai et al. (102) investigated the effects of early Cu exposure on gut microbiota and its metabolites in Sprague–Dawley rats. The results indicated that copper exposure exhibited dose-dependent effects on α and β-diversity of gut microbiota, reducing the relative abundance of Firmicutes and Bacteroidetes, beneficial bacteria abundance, as well as bacteria associated with lipid metabolism and enteritis. For example, Cu exposure caused a decrease in the abundance of Lactobacillus, Bifidobacterium, and Romboutsia, which are common probiotics involved in carbohydrate metabolism (118). Romboutsia, a newly discovered gut probiotic, participates in various metabolic processes such as carbohydrate utilization, amino acid fermentation, and anaerobic respiration. Liao et al. found that excess Cu had significant effects on the microbiota and metabolites in the jejunum and colon, which were involved in intestinal barrier dysfunction and inflammation (119). Cu exposure can alter the metabolic activities of the gut microbiota, such as protein metabolism and amino acid biosynthesis, which may be related to metabolic disorders of the host. A study reported that organic and inorganic Cu sources had distinct consequences on the composition of the cecal microbiota and short-chain fatty acids (SCFAs) in rabbits. Specifically, the CuCit group demonstrated a higher relative abundance of the genera Rikenella and Lachnospiraceae\_NK3A20\_group, which could potentially contribute to the reduced incidence of diarrhea in rabbits (120). Metabolomic analysis of fecal microbiota also confirmed the impact of early Cu exposure on pathways related to liver damage and intestinal inflammation. Furthermore, another study found that high levels of copper in the diet (120 and 240 mg/kg) altered the quantity and diversity of microbiota in Sprague–Dawley rats’ feces, accompanied by elevated serum tumor necrosis factor α (TNF-α) levels (121). These studies suggest that excessive Cu exposure can lead to liver damage and gut microbiota dysbiosis in rats, disrupting intestinal barrier function and affecting various metabolic pathways (118). Cu exposure decreased the abundance of bacteria capable of producing short-chain fatty acids (SCFAs) in juvenile common carp, such as Blautia, Faecalibacterium, Roseburia, Ruminococcus, and Lachnospiraceae (104). The abundance of Roseburia was positively correlated with the levels of lipogenic enzymes, total protein, and triglycerides, and negatively correlated with lipolytic enzyme levels. Copper exposure disrupted gut microbiota associated with lipid metabolism and immunity in freshwater fish, increasing the risk of pathogen invasion (104). Adding Cu to the diet reduced the relative abundance of butyrate-producing bacteria in the intestine, such as Faecalibacterium, Roseburia, and Bacteroides. Metabolomic analysis has shown that dietary Cu levels affect protein and carbohydrate metabolism, protein biosynthesis, urea cycle, gluconeogenesis, and amino acid metabolism (including arginine, proline, β-alanine, phenylalanine, tyrosine, and histidine metabolism) (96).

### 4.2 Manganese (Mn)

Manganese is an essential trace element required for amino acid, lipid, and carbohydrate metabolism (122). It can penetrate the blood–brain barrier, accumulate in the brain, and lead to neurodegenerative diseases such as Parkinson’s disease (PD) (123). Growing evidence has revealed that Mn is important for maintaining bacterial fitness and survival. It can alter DNA replication activities in bacteria by serving as a cofactor for ribonucleotide reductase, which is involved in DNA synthesis from RNA (124). In addition to its function as an enzyme cofactor, studies have shown that Mn promotes antioxidative effects in bacteria independent of protein activity. Mn also protects bacteria against oxidative stress by modulating the Dps protein, resulting in DNA binding, iron sequestration, and inhibition of ferroxidase activity (99). Mn exposure leads to a decrease in the abundance of gut microbiota in rats, especially Prevotellaceae, Lachnospiraceae, and Lactobacillaceae, and alters the metabolism of serotonin, deoxycholic acid, beta-hydroxybutyrate, and uric acid (107). The regulatory effect of Mn on the gut microbiota has also been confirmed in non-mammals. Studies have shown that during the laying period, supranutritional doses of Mn (30 mg/kg Mn, sustained for 10 weeks) increased the abundance of Prevotellaceae, Bacteroidetes, and Ruminococcaceae in the goose intestine while reducing the abundance of Streptococcaceae, simultaneously improving the hatchability and eggshell thickness of hatched eggs (108). Due to the high metabolic activity of the gut microbiota, interference by Mn with the gut microbiota leads to significant changes in gut metabolomics. Specifically, preliminary data suggest that oral administration of excess Mn (15 mg/kg/day, administered orally by gavage for 30 days) affects fecal metabolomics, including decreased levels of fecal butyrate, α-tocopherol, and bile acids and increased levels of palmitic acid and bile acids in rat feces, which may partially mediate the effects of Mn in the body (125). The gut microbiota plays a crucial role in the development of the nervous system through the gut-brain axis. Studies have found that Mn exposure increases the generation of beta-amyloid protein (Aβ) and tau protein in the brains of rats, causing hippocampal degeneration and necrosis. Manganese exposure induces neurotoxicity by increasing inflammation in the peripheral blood and central nervous system. Transplanting normal rat fecal microbiota into rats exposed to Mn can alleviate Mn-induced neurotoxicity (109). In addition, animal studies have shown that Mn can affect the biodiversity of the gut microbiota in mice, and this effect exhibits some gender specificity. For example, the response of female mice to oral Mn exposure resulted in a significant decrease in the abundance of Firmicutes and an increase in the abundance of Bacteroidetes. In contrast, excess Mn was associated with a significant decrease in the relative abundance of Bacteroidetes and a significant increase in the relative abundance of Firmicutes in male mice. Additionally, the observed changes in the above-mentioned microbial populations in female mice were related to the significant upregulation of genes associated with LPS biosynthesis. Mn exposure also significantly affected the levels of gamma-aminobutyric acid (GABA) and glutamate produced by intestinal bacteria in the fecal samples. Metabolomic analysis of feces revealed that Mn exposure resulted in both the upregulation and downregulation of precursors of neurotransmitters or neurotransmitter synthesis, such as glycine, glutamate, and phenylalanine, in female and male mice, respectively. These findings suggest that manganese-induced dysbiosis of the gut microbiota may disrupt the normal metabolism of neurotransmitters or related precursors in the intestine, thereby further interfering with normal gut-brain axis function (106).

### 4.3 Iron (Fe)

Iron is a crucial component of hemoglobin and myoglobin and participates in various physiological processes in organisms, especially in DNA synthesis, red blood cell production, oxygen transport, energy metabolism, and enzyme activity. Iron is absorbed in the form of Fe^2+^ in the duodenum and small intestine, where it binds to transferrin after absorption to form ferritin, and Fe^2+^ is converted to Fe^3+^ within ferritin. Transferrin transports iron to multiple organs, including the liver, where it is stored as ferritin (126). Most bacteria in the intestine require iron to maintain normal growth and survival. Iron alters the intestinal microbiota by regulating microbial acquisition of energy from host-ingested nutrients (100). Both commensal and pathogenic bacteria acquire dietary iron in the intestine through at least two pathways (1): receptor-mediated transport of iron-binding proteins (i.e., transferrin and heme) and (2) release of iron from iron carriers. Studies have shown that iron transport proteins such as iron uptake systems (FeoAB) and iron transport systems (SitABCD) are expressed in several bacteria, including *Salmonella enterica* (127). Iron deficiency can modulate mucosal immunity, at least in part by changing the microbial profile and function (113). Iron deficiency alters the microbiota in pig intestines, leading to increased levels of volatile fatty acids (VFAs), including acetate, propionate, and butyrate (113). VFAs produced by gut microbiota, such as propionate, regulate iron absorption as a compensatory mechanism for low iron levels. These findings suggest that the gut microbiota plays a critical role in the effects of iron on both gut immunity and brain development. Besides, low-iron diets have been shown to promote an increase in the abundance of Lactobacillus, a decrease in the abundance of Fibrobacteres, and a decrease in the number of opportunistic *Escherichia coli*-Shigella in pig intestines, leading to a decrease in the proinflammatory cytokine tumor necrosis factor α (TNF-α) levels, thereby improving intestinal function, reducing the incidence of diarrhea, and promoting pig growth (111).

High-iron diets have been found to reduce the relative abundance of beneficial bacteria (Akkermansia, Bifidobacterium, and Lactobacillus) in mouse intestines, while increasing the relative abundance of pathogenic bacteria (Romboutsia and *Clostridium difficile*), leading to significantly decreased expression of genes regulating fat formation and adipocyte differentiation in the liver and adipose tissue of mice, with significantly increased expression of lipolytic enzyme-related genes. These studies indicate that a high-iron diet reduces lipid deposition by inhibiting fat formation and promoting fat breakdown (110). Compared to the normal iron diet group, the iron overload group showed a significant increase in the abundance of Allprevotella, Eubacteriumcoprostanoligenes, and Muribaculum, while the abundance of Dubosiella, Lactobacillus, and Bifidobacterium significantly decreased. Iron overload reduces the levels of anti-inflammatory metabolites [such as (+)-α-tocopherol] in the mouse intestine. Additionally, both iron deficiency and iron overload resulted in decreased nucleotide and enzyme metabolism levels and increased lipid metabolism levels in mouse intestines, suggesting that iron overload exacerbates colitis in mice by regulating iron death and disrupting gut microbiota composition (112). Early iron status affects the composition of the microbial community and the level of VFAs in pig intestines; however, these differences are largely normalized after subsequent dietary iron supplementation (113).

### 4.4 Other trace elements and gut microbiota

Some trace elements commonly found in the environment lie between toxic heavy metals and essential trace elements, such as chromium (Cr), silicon (Si), nickel (Ni), and others, exhibiting different effects on gut microbiota depending on their different doses and forms. The interactions between these metals and gut microbiota might be positive or negative according to the different valence states, concentrations, and forms of the same heavy metal. Recently, there have been few studies on the relationship between the above trace elements and gut microbiota.

#### 4.4.1 Chromium (Cr)

Cr primarily exists in two stable forms: Cr^3+^ and Cr^6+^ (128). Trivalent chromium (Cr^3+^) is an essential trace element for the growth and development of livestock and poultry and its addition to feed can improve animal productivity and feed efficiency (129). Chromium picolinate is a common dietary supplement, with Cr^3+^ enhancing insulin activity and reducing the risk of diabetes (130). Conversely, hexavalent chromium (Cr^6+^) is toxic and leads to liver and kidney damage, and respiratory diseases (131). It is generally believed that the toxicity of Cr^6+^ to aquatic organisms is greater than that of Cr^3+^ (132). At present, there are few studies on the effects of Cr on the gut microbiota, especially Cr^3+^. Only one study found that 0.4 mg/kg chromium supplementation can influence the composition of gut microbiota of heat-stressed broilers. The results showed that Cr improved performance, insulin resistance, and sodium-glucose transporter 1 expression, and altered gut microflora structure and secretion of gastrointestinal peptides, thus showing that supplementation with chromium is beneficial for maintaining glucose homeostasis and alleviating heat stress (133).

Cr and its compounds are widely used in industries such as printing and dyeing, leather production, and metallurgy. They are inevitably released into the environment and pose a significant threat to living organisms (132). Research has shown that Cr^6+^ may lead to various adverse outcomes by affecting the structure and metabolites of the gut microbiota (Table 3).

**Table 3:** The relationship of other heavy metals and gut microbiota as well as heavy metal-induced changes in metabolites.

Heavy metals	Experiment model	Effect on gut microbiota composition	Effect on gut metabolite and gut barrier	Outcome	References
Cr	Zebrafish	↑ Actinomycetes, Acinetobacter, Acidophorax, Mycobacterium, Aeromonas, Hydrophagophaga, Brevundimonas ↓ Bacteroidetes, Chryseobacterium, Pseudomonas, Delftia, Ancylobacter	Disturb cholesterol metabolism, including primary bile acid and microbiota-related secondary bile acid metabolism	Swimming distance and locomotor behavior was decreased Acetylcholinesterase activity was reduced in Cr-exposed groups	(135)
Cr	Chickens	↑ Bacterial taxa including 3 phyla and 17 genera ↓ Bacterial taxa including 2 phyla and 47 genera	The gut microbial alpha-diversity decreased	Decreased the gut microbial diversity and altered microbial composition of chickens	(151)
Cr	Grouper	↑ Proteobacteria, Firmicutes, Actinobacteria, Chloroflexi, Bacteroidetes ↓ Solimonadaceae, Actobacillales and KD3-93	The alpha diversity of intestinal microbiota communities was decreased.	The relative abundance of metabolic pathway and immune-related pathway were significantly decreased	(134)
Cr	Mouse	↑ Verrucomicrobia, families Akkermansiaceae, Saccharimonadaceae, genus Akkermansia ↓ Firmicutes and Bacteroidetes, SCFA-producing bacteria	Cr (VI) significantly decreased DMH-induced SOD, GSH and CAT levels, while, the MDA level increased.	Cr (VI) increased weight loss in DMH-induced mice Promoted the formation of tumors	(136)
Cr	*Bufo gargarizans* tadpoles	↑ Proteobacteria ↓ Aeromonas, Fimicutes, Bacteroidetes	The diversity of gut microbial community was significantly decreased	Carbohydrate metabolism, amino acid metabolism, energy metabolism, metabolism of cofactors and vitamins, nucleotide metabolism, xenobiotics biodegradation metabolism and lipid metabolism were significantly increased Elevated risk of disease, such as neurodegenerative diseases, immune system diseases, metabolic diseases, infectious diseases and cancers	(137)
Ni	Occupational workers	↑ Parabacteriadies, Escherichia-Shigella ↓ Lactobacillus, Lachnospiraceae_Unclassfied. Blautia	Impaired intestinal degradation of purines and upregulated biosynthesis of primary bile acids	Serum uric acid elevation	(140)
Ni	Mice	↑ inflammation-related taxa Alistipes and Mycoplasma ↓ Lactobacillus, Blautia	Purine nucleosides were accumulated in mice feces,	Promotes uric acid elevation and systemic inflammation	(140)
Ni	Mice	↑ Bacteroides, Intestinimonas ↓ Lachnospiraceae_NK4A136_group Lachnospiraceae_UCG-001_group, ratio of Firmicutes/Bacteroides	_	No statistical difference in body animal weight was found	(141)
Ni	Fish	↑ Proteobacteria ↓ Lactobacillus	Diversity of the gut microbiome was significantly reduced	Transcription of cytokines IL-1, IL-10, and TNF-a was significantly upregulated, IL-7 was significantly downregulated	(141)
Cr-Ni	Mice	↑ Patescibacteria, Parasutterella, Alistipes, Parabacteroides ↓ Firmicutes and Verrucomicrobia, Lactobacillu and Akkermansia	These differential metabolites were significantly enriched in signaling pathways such as amino acid metabolism (tryptophan and lysine, etc.), lipid metabolism (arachidonic acid), the immune system (Fc gamma R-mediated phagocytosis), and apoptosis	Resulting in an imbalance of the intestinal flora and disorders of metabolites and metabolic pathways	(143)
Si	Rats and mice	↑ Ruminococcaceae UCG-005, Prevotellaceae NK3B31, *Weissella paramesenteroides*, *Lactobacillus reuteri*, *Lactobacillus murinus* ↓ Mucispirillum, Rodentibacter, and *Staphylococcus aureus*	BT drinking significantly suppressed pylorus ligation enhanced gastric juice secretion, gastric reactive oxygen species amount, erythrocyte extravasation, IL-1β production by infiltrating leukocyte, and lipid peroxidation within gastric mucosa.	BT drinking did not affect the body weight but significantly reduced the weight of feces and gastrointestinal motility	(147)
Si	Mice	↑Firmicutes and Patescibacteria	_	Significantly caused the spatial learning and memory impairments and locomotor inhibition	(148)
Si	Rats	At different stages, the changes of intestinal microflora in rats were different after silica exposure.	Serum metabolites and disrupted multiple metabolic pathways differed after varied silica exposure exposure duration.	Time-specific changes in crosstalk among cytokines, the gut microbiota, and metabolites may be a potential mechanism for silica-induced lung injury	(149)
Si	Mice	β-diversity was dose-dependently disrupted	Bacterial SCFAs were dose-dependently reduced after the administration of SiO2and TiO2NPs.	Dose-dependent decreases in the expression of IL-6 in the liver, circulating TG and BUN Atheromatous disease or glucose intolerance after NP exposure was not observed	(152)

↑, higher/increased; ↓, lower/decreased; —, Not applicable; Cr, Chromium; Ni, Nickel; Si, silicon; DMH, 1, 2-dimethylhydrazine; MDA, malondialdehyde; CAT, catalase; GSH, glutathione; SOD, superoxide dismutase; TNF, tumor necrosis factor; IL, interleukin; BT, silicon-containing water; TG, triglyceride; SCFAs, short chain fatty acids; BUN, urea nitrogen; NPs, nanoparticle.

Ye et al. (134) found that short-term Cr exposure reduced the diversity of intestinal microbial communities, resulting in significant changes in the intestinal microbial structure of fish, with Proteobacteria, Firmicutes, Actinobacteria, Bacteroidetes, and Cyanobacteria being the major phyla. Conversely, at the genus level, the abundance of Nitrosomonas, Planococcaceae, Bacillus, and Spirochaeta significantly decreased (134). Cr exposure reduced the swimming distance and locomotor behavior of zebrafish larvae, decreased acetylcholinesterase activity, and reduced total cholesterol levels. The differential expression of metabolites induced by chromium exposure mainly enriched primary bile acid biosynthesis, indicating that chromium exposure may promote cholesterol conversion. Compared to the control group, the Cr^6+^ exposure group showed a decrease in the abundance of Proteobacteria and an increase in the abundance of Actinobacteria in the zebrafish larval intestines. At the genus level, the abundance of Pseudomonas, Acidovorax, Burkholderia, Aquabacterium, Shewanella, and Shigella increased, whereas the abundance of Flavobacterium, Pseudorhodoferax, Delftia, and Anaerolinea decreased. Further analysis of the correlation between the gut microbiota and bile acid metabolites showed that changes in the gut microbiota induced by chromium exposure may be related to secondary bile acid metabolism. Overall, Cr exposure may disrupt cholesterol metabolism, including primary bile acid and microbiota-associated secondary bile acid metabolism (135). Zhang et al. found that Cr^6+^ regulated the structure of the intestinal microbiota and metabolites in 1,2-dimethylhydrazine (DMH)-induced colorectal cancer mice, resulting in weight loss in DMH-induced mice and promoting tumor formation (136). Yao et al. found that Cr^6+^ exposure led to a decrease in gut microbiota diversity and significant changes in the structure of tadpoles, potentially further increasing the risk of opportunistic pathogen infection and intestinal disease (137).

#### 4.4.2 Nickel (Ni)

Ni is a common heavy metal in nature and is essential for plant growth. Modern medicine often utilizes nanotechnology, such as nickel nanoparticles (NiNPs), as carriers for drug delivery (138). Ni can cause various adverse effects on human health, including allergies, cardiovascular and kidney diseases, pulmonary fibrosis, and cancer (139). Currently, there is limited research on the relationship between Ni and the gut microbiota. Studies have focused on the detrimental effects of nickel on intestinal flora, causing intestinal dysbiosis. However, other studies have shown that a low-Ni diet may improve intestinal dysbiosis. Yang et al. found that long-term exposure to Ni in occupational workers led to a decreased abundance of Lactobacillus, Lachnospiraceae_ Unclassified, and Blautia in the gut, while pathogenic bacteria including Parabacteroides and Escherichia-Shigella increased in abundance. Gut purine degradation was impaired, and endogenous bile acid biosynthesis was upregulated. Consistent with the results in humans, the mouse experimental results showed that Ni exposure significantly promoted hyperuricemia and systemic inflammation. Under Ni treatment, lactobacilli and Bacteroides in the mouse gut microbiota decreased, whereas inflammation-related taxa, such as Alistipes and Spirochaeta, increased. Additionally, metabolomic analysis showed that purine nucleosides accumulated in mouse feces and Ni exposure increased purine absorption and serum uric acid (140). Zhou et al. found that the oral administration of 400 μM NiSO_4_·6H_2_O was non-toxic to mice (no statistical difference in body weight was observed between the Ni-exposed and control groups). However, at the genus level, the relative abundances of Bacteroides and Intestinimonas were significantly higher in the oral nickel group than in the control group, whereas the relative abundances of Lachnospiraceae_NK4A136_group and Lachnospiraceae_UCG-001_group were significantly lower than those in the control group. Furthermore, the Firmicutes/Bacteroidetes ratio in the Ni-exposed group was significantly lower than that in the control group, indicating that Ni may affect the structure and composition of the mouse gut microbiota but has no effect on body weight (141). However, another study confirmed that a low-nickel diet had certain benefits in improving intestinal dysbiosis in patients with systemic nickel allergy syndrome (SNAS), and strongly supported the use of appropriate probiotic supplementation to significantly improve dysbiosis or restore healthy microbial populations in the gut (142). The pollution and toxicity of hexavalent chromium Cr^6+^ and nickel Ni^2+^ have become a global public health issue. Long-term exposure to Cr and Ni can disrupt the balance of the gut microbiota. Gu et al. found that chronic exposure to Cr^6+^, Ni, or their combination altered the composition of the mouse colon microbiota, resulting in dysbiosis of the gut microbiota and disruption of metabolites and metabolic pathways, thereby significantly affecting the inflammatory response in animals. Moreover, compared with exposure to individual heavy metals, chronic combined exposure to Cr-Ni alleviated intestinal barrier damage and the inflammatory response by reducing trace element levels and the expression levels of inflammatory factors, indicating an antagonistic effect between Ni and Cr (143).

#### 4.4.3 Silicon (Si)

Si is an essential trace element in the human body and the third most abundant trace element in the human body. Naturally occurring silicon in food exists in several forms including silicon dioxide (SiO_2_), free orthosilicic acid (H4SiO_4_), and silicic acid bound to certain nutrients (144). Silicon plays a significant role in human health, including bone mineralization, collagen synthesis, anti-aging, regulation of antioxidant enzymes, and reduction in the risk of atherosclerosis and neurological diseases (145). Animal experiments have shown that silicon may be involved in antioxidant defense and anti-apoptotic pathways (146). Drinking silica-containing water increased the abundance of beneficial bacteria and decreased the abundance of harmful bacteria. Research has found that drinking silicon-containing water increases the abundance of beneficial bacteria, such as *Weissella paramesenteroides*, *Lactobacillus reuteri*, and *Lactobacillus murinus*; reduces the abundance of harmful bacteria, such as *Staphylococcus aureus*; and significantly inhibits the increase in free radicals, extracellular leakage of red blood cells, and gastric mucosal damage caused by gastric juice accumulation, leading to elevated lipid peroxidation marker 4-HNE and proinflammatory cytokine IL-1β (147). This study provides scientific evidence for the potential effects of water-soluble silicon intake on antioxidant activity, gastrointestinal function, and modulation of the gut microbiota in mice (144). However, another study showed that oral administration of the commonly used food additive silicon dioxide nanoparticles (SiO_2_NPs) enhanced microbial diversity in the mouse intestine, mainly manifested as an increased abundance of Firmicutes and Patescibacteria, leading to spatial learning and memory impairments and motor inhibition in mice. The neurotoxic effects induced by SiO_2_NPs may occur through a unique gut-brain axis (148). Guo et al. found that lung injury caused by different durations of silica exposure might affect serum metabolism and disrupt gut microbiota through pulmonary inflammation (149).

## 5 Conclusion and future perspectives

This review summarizes the current understanding of the relationship between 10 common heavy metals and gut microbiota, as well as their metabolic products and health implications. The results demonstrated a bidirectional relationship between heavy metal or trace element exposure and gut microbiota. Disruption of the gut microbiota by heavy metal exposure can affect its metabolism and physiological functions, partially leading to the etiology or progression of metabolic diseases. Deficiency or excess of essential trace elements, such as iron, manganese, and copper, can also lead to metabolic disorders, overgrowth of pathogenic bacteria, and immune dysfunction. The interaction between bacteria and metals is influenced by factors such as dose, duration, mode of ingestion/administration, metal species, and bioavailability. The majority of animal studies conducted thus far have primarily focused on a single metal, with only a few studies examining multiple combinations. In contrast, real-life exposure typically involves exposure to multiple metals. In particular, joint exposure to ubiquitous and mildly toxic microplastics and heavy metals is crucial for the combined effect on the gut microbiota. Additionally, exposure to mixtures of heavy metals may have additive, synergistic, or antagonistic effects on the gut microbiota. Furthermore, gender is a specific factor in the effect of heavy metals on the gut microbiota. The structure and composition of the gut microbiota are related to host gender, and gender differences in the host gut microbiota can drive the regulatory effects of hormones on autoimmune diseases (150). In view of this, future research directed to multi-metal combination and gender-specific impact on gut microbiota. In addition, feasible and efficient probiotic strategies and biomarker development are required to understand the disruption and health implications of the intestinal microbiota caused by heavy metals.

## Author contributions

QZ: Conceptualization, Methodology, Project administration, Writing – original draft, Writing – review & editing, Investigation. BC: Data curation, Methodology, Validation, Writing – original draft. FZ: Investigation, Validation, Writing – original draft. BZ: Investigation, Validation, Writing – original draft. YG: Investigation, Validation, Writing – original draft. MP: Resources, Supervision, Validation, Writing – review & editing. LH: Resources, Supervision, Validation, Writing – review & editing. TW: Investigation, Methodology, Project administration, Resources, Supervision, Writing – review & editing.
